# Detection of Systemic Canine Kobuvirus Infection in Peripheral Tissues and the Central Nervous System of a Fox Infected with Canine Distemper Virus

**DOI:** 10.3390/microorganisms9122521

**Published:** 2021-12-06

**Authors:** Franziska K. Kaiser, Lydia van Dyck, Wendy K. Jo, Tom Schreiner, Vanessa M. Pfankuche, Peter Wohlsein, Ilka Baumann, Martin Peters, Wolfgang Baumgärtner, Albert D. M. E. Osterhaus, Martin Ludlow

**Affiliations:** 1 Research Center for Infectious Disease and Zoonoses, University of Veterinary Medicine Hannover, 30559 Hannover, Germany; franziska.kaiser@tiho-hannover.de (F.K.K.); wendy.karen.jo@tiho-hannover.de (W.K.J.); ilka.baumann@tiho-hannover.de (I.B.); albert.osterhaus@tiho-hannover.de (A.D.M.E.O.); 2 Department of Pathology, University of Veterinary Medicine Hannover, 30559 Hannover, Germany; lydia.van.dyck@tiho-hannover.de (L.v.D.); tom.schreiner@tiho-hannover.de (T.S.); vanessa.pfankuche@tiho-hannover.de (V.M.P.); peter.wohlsein@tiho-hannover.de (P.W.); wolfgang.baumgaertner@tiho-hannover.de (W.B.); 3 Chemisches und Veterinäruntersuchungsamt Westfalen, 59821 Arnsberg, Germany; martin.peters@cvua-westfalen.de

**Keywords:** canine kobuvirus, canine morbillivirus, *Vulpes vulpes*, next-generation sequencing, extraintestinal kobuvirus

## Abstract

Canine kobuvirus (CaKV) is a globally distributed pathogen of dogs and is predominantly associated with infection of the gastrointestinal tract. However, an etiological link to enteric disease has not been established since CaKV has been identified in both asymptomatic dogs and animals with diarrheic symptoms. In this study, an extraintestinal CaKV infection was detected by next-generation sequencing in a fox (*Vulpes vulpes*) in Germany concomitant with a canine distemper virus (canine morbillivirus; CDV) co-infection. Phylogenetic analysis of the complete coding region sequence showed that this strain was most closely related to a CaKV strain detected in a dog in the United Kingdom in 2008. The tissue and cellular tropism of CaKV was characterized by the detection of viral antigens and RNA. CaKV RNA was detected by in situ hybridization in different tissues, including epithelial cells of the stomach and ependymal cells in the brain. The use of a new RT-qPCR assay for CaKV confirmed the systemic distribution of CaKV with viral RNA also detected in the lymph nodes, bladder, trachea, and brain. The detection of a CDV infection in this fox suggests that immunosuppression should be further investigated as a contributing factor to the enhanced extraintestinal spread of CaKV.

## 1. Introduction

Canine kobuvirus (CaKV) is a non-enveloped RNA virus containing a single-stranded, positive-sense RNA genome and is a member of the species Aichivirus A in the genus Kobuvirus of the family Picornaviridae [1,2]. The genome of CaKV consists of 8.1–8.2 kbp encoding a 2469 amino acid polyprotein, which is comprised of a leader protein, three structural proteins (VP0, VP3 and VP1), seven non-structural proteins (2A, 2B, 2C, 3A, 3B, 3C, and 3D), and is flanked by untranslated regions [3,4]. The six officially recognized viruses that are included within the species Aichivirus A include human Aichivirus [5,6], CaKV [7], murine kobuvirus [8], Kathmandu sewage kobuvirus [9], roller kobuvirus [10], and feline kobuvirus [11]. Phylogenetic analysis has shown a close relationship between human Aichivirus and CaKV [4]. This is demonstrated by the discovery of a novel kobuvirus in Japanese sewage systems which has 83% nucleotide similarity to the human AiV A846/88, but 95% with the canine CaKV strain US-PC0082 [12], which raises questions about the potential for interspecies and zoonotic virus transmission.

CaKV was first detected in dogs in the United States of America [7] and has been subsequently identified in domestic dogs worldwide. The transmission of CaKV has also been reported in wild carnivore species including foxes (*Vulpes vulpes*) [13] and wolves (*Canis lupus*) in Italy [14], golden jackal (*Canis aureus*), side-striped jackal (*Canis adustus*), and spotted hyena (*Crocuta crocuta*) in Tanzania [15], and is endemic globally [3,16,17,18]. The high reported seroprevalence of 37.4% in dogs suggests that exposure to this virus is frequent in European pet dogs [19]. However, the cellular and tissue tropism of CaKV is unknown.

The postulated link between CaKV infection and clinical gastrointestinal disease remains unproven, as this virus has been detected in cases of diarrhea but is equally present in asymptomatic animals [3,20]. Similar observations have been made in cases of other mammalian kobuvirus infections, in which the virus was identified in clinically healthy individuals [21,22,23], and also in association with gastroenteritis disease of varying severity [24,25,26,27]. Subsequent studies indicate a key role for concomitant infections in kobuvirus positive and diarrheic dogs [16,28] with CaKV characterized as a potential secondary infection [18]. In particular, CaKV infections have been associated with cases of coinfection with viruses associated with immunosuppression, such as canine distemper virus (CDV) [29] or canine parvovirus [16,28].

## 2. Materials and Methods

### 2.1. Post-Mortem Examination

The animal described in this study is an adult male fox (*Vulpes vulpes*) which was found dead in North Rhine-Westphalia, Germany, in 2016, and submitted to the Chemical and Veterinary Investigation Office (CVUA) Westfalen located in Arnsberg, Germany. A comprehensive post-mortem examination was conducted, and organ samples were harvested for use in bacteriological investigations and stored frozen at −80 °C for later analysis in molecular biological diagnostic assays. For further histopathological diagnostic purposes, tissue samples were fixed in 10% neutral-buffered formalin and embedded in paraffin wax according to standard procedures prior to immunohistochemical staining and in situ hybridization.

### 2.2. Next-Generation Sequencing and Analysis

Sample preparation of tonsillar tissue, nucleic acid extraction with TRIzol^TM^ Reagent (Fisher Scientific), and viral enrichment prior to next-generation sequencing was performed as described previously [30]. The Nextera XT DNA Sample Preparation Kit protocol (Illumina, San Diego, CA, USA) was used for library preparation. Sample libraries were sequenced on an Illumina MiSeq platform with a MiSeq Reagent Kit v3 (300 x 2 cycles). Analysis of the quality-trimmed reads was performed by de novo assembly of contigs larger than 500 bp using CLC Genomics Workbench 9.0 (Qiagen GmbH, Hilden, Germany). BLAST analysis was conducted with the obtained contigs to identify the presence of viral sequences. This enabled a complete genome sequence of CDV and an almost complete sequence of CaKV to be recovered. The complete CaKV genome sequence was obtained by the use of a standard gap filling RT-PCR protocol. The identity of two sequence gaps (3626–3673 bp and 2591–2686 bp) was determined by Sanger sequencing following the amplification of amplicons spanning the sequence gaps through the use of the Phusion^®^ High-Fidelity PCR kit (NEB, Ipswich, MA, USA) with the primer pairs CaKV_2521-2539_Fw (5′-acgaYtggctYgaRttcgc-3′), CaKV_3016-3033_Rv (5′-ggacgttRaaggaYtcgc-3′) and CaKV_3479-3498_Fw (5′-gctBtccaaYttcttcatgg-3′), CaKV_3894-3912_Rv (5′-cataggtgggYctatctgc-3′). Additionally, a rapid amplification of cDNA ends (RACE) protocol was used to amplify and sequence the authentic 5′ genome end (1–155 bp) using a rapid amplification of cDNA ends (RACE) primer 5′-AATTCACTGACGGGTCTAGC-3′), as described previously [31]. The phylogenetic relationship of the CaKV was determined by an alignment of the newly generated kobuvirus sequence and available genomes deposited in GenBank and the genome sequence generated in this study (GenBank accession no. MN337880) by using MAFFT alignment version 7 [32]. The resulting alignment was used in MEGA 7 [33] to construct a phylogenetic tree using the maximum likelihood method with GTR+G used as the best-fit model of nucleotide substitution. All phylogenetic trees were tested by bootstrapping with 1000 replicates.

### 2.3. Immunohistochemistry and In Situ Hybridization

Tissue sections from peripheral tissues or the central nervous system (CNS) were in-vestigated by immunohistochemistry for the presence of the CDV antigen. Immunohistochemistry was performed as described previously using the monoclonal antibody D110, which is directed against the CDV nucleoprotein and the avidin–biotin–peroxidase complex method [34]. The tissue distribution and cellular tropism of CaKV was investigated using in situ hybridization for CaKV as formerly described with self-designed, digoxigenin (DIG)-labeled sense and anti-sense probes (probe sequence: 5’GGAATGACCGTCGTCTCAACGATGGCGTAAACCTCGACACCCAACTCTTCCTCAAACACGCAAAGGTGATCAGACCGAGCC 3´; forward primer used for probe design: GGAATGACCGTCGTCTCAAC; reverse primer used for probe design: GGCTCGGTCTGATCACCTTTG).

### 2.4. Virus Detection by RT-PCR and RT-qPCR

A systemic CDV infection was verified by reverse transcription PCR (forward primer ACAGGATTGCTGAGGACCTAT, reverse primer CAAGATAACCATGTACGGTGC [35]), in multiple tissue types of the infected fox by a OneStep RT-PCR Kit (Qiagen, Hilden, Germany). Screening of different organ material for CaKV RNA was performed using a newly developed virus-specific reverse transcription-quantitative PCR (RT-qPCR). Oligonucleotides were designed to target a 120 bp region (6764–6883 bp) of the CaKV genome. The resulting primers (forward primer 5′-atgaccgYcgtctcaaYgRtgg-3′, reverse primer 5′-tRgaRaagtagaggtcagcagc-3′) and the probe (5′-FAM-ttcctcaaacacggcaaaggtgatcagacc-BHQ1-3′) were then used in a 45-cycle one-step qRT-PCR with an annealing temperature of 57 °C and a Luna Probe One-Step RT-qPCR kit (NEB, Ipswich, MA, USA) according to the recommended protocol. To prepare the nucleic acids of the available samples for the PCR assays, organ tissue was homogenized using a FastPrep-24 5G homogenizer (MP Biomedical, Irvine, CA, USA), centrifuged (12,000× *g* for 5 min), and the supernatant used for RNA extraction with TRIzol^TM^ Reagent (Thermo Fisher Scientific, Waltham, MA, USA) following the manufacturer’s instructions.

## 3. Results

### 3.1. Macro- and Histopathology Findings

**Figure 1:**
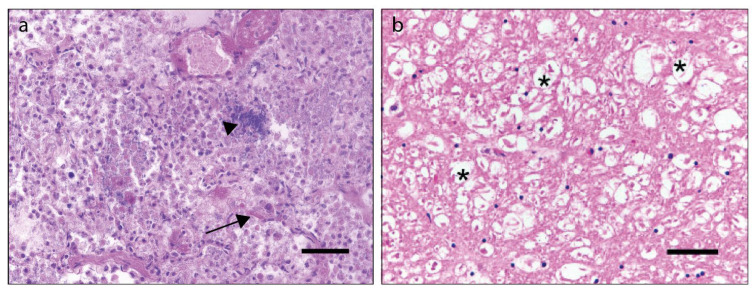
Histological analysis of hematoxylin- and eosin-stained sections of formalin-fixed paraffin-embedded fox tissues (**a**) Severe, purulent, and necrotizing pneumonia in the lung with multifocal colonies of coccoid bacillae (arrowhead) and microthrombosis (arrow). (**b**) A hematoxylin- and eosin-stained section of the cerebrum of a fox shows a multifocal vacuolization of the white matter (stars). Scale bar (**a**,**b**), 50 µm.

The carcass of the adult male fox was in a bad nutritional condition at the timepoint of necropsy and a catarrhal enteritis and purulent eye discharge were observed. Bacteriological investigation resulted in the isolation of *Steptococcus canis*, *Staphylococcus pseudintermedius*, and *Streptococcus dysgalactiae equisimilis* from lung tissue with evidence of macroscopic pathology. In addition, there was a mild depletion of the white pulp of the spleen and nematodes were present in the urinary bladder, presumably representing *Pearsonema plica* [36]. Histological examination showed intracytoplasmic, eosinophilic inclusion bodies in the bronchial epithelium and the epithelium of the urinary bladder. A lobular, severe, purulent, and necrotizing pneumonia was also present with multifocal colonies of bacteria and a mild interstitial pneumonia (Figure 1a). Furthermore, multifocal vacuolization of the white matter was observed in the cerebrum, cerebellum, and brain stem (Figure 1b).

### 3.2. Virus Detection and Phylogenetic Analysis

**Figure 2:**
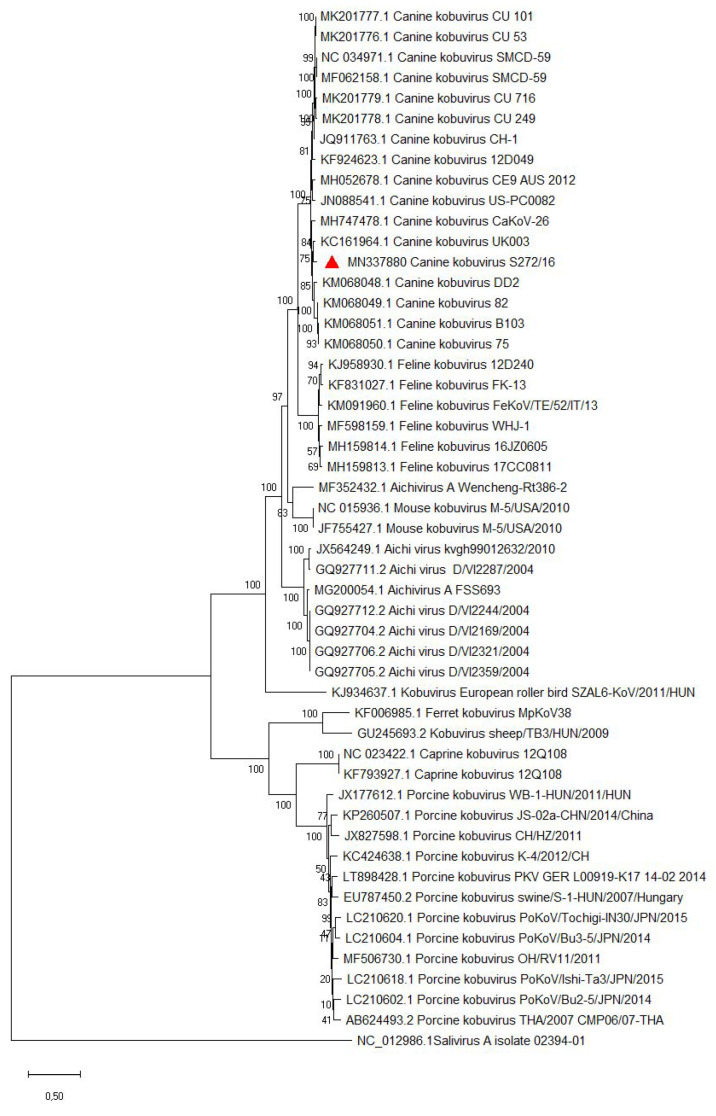
A Bayesian phylogeny tree based on the complete coding region sequences of kobuviruses. An isolate of Salivirus A (GenBank accession no. NC_012986.1) was used as an outgroup. The canine kobuvirus sequence analysed in this study (GenBank accession no. MN337880) is indicated with a red triangle. Numbers at the nodes indicate posterior probabilities percentage. GenBank accession numbers are provided for comparison isolates. Scale bars indicate nucleotide substitutions per site.

Tonsillar tissue from the fox under investigation was used as sample material for NGS to identify any viruses which could have contributed to the observed macro- and histopathology. Analysis of the resulting NGS reads using CLC Genomics Workbench 9.0 (Qiagen GmbH, Hilden, Germany) enabled the recovery of a complete CDV genome sequence and a partial CaKV genome sequence which was completed using gap filling and RACE protocols. In recent years, an increasing number of full genome sequences of CaKV strains have been published. To investigate the evolutionary origins of the complete CaKV genome sequence generated in this study (8312 bp; GenBank accession no. MN337880), a phylogenetic analysis was performed with CaKV sequences downloaded from GenBank which contain the complete coding region. This showed that the CaKV strain (S272/16) present in the German fox showed a close genetic identity of 94.79% to a CaKV strain from the UK (KC161964.1; Figure 2). In addition to the CaKV sequence, a complete CDV genome sequence (15690 bp) (GenBank accession no. MN267061) was obtained by metagenomic analysis. This particular CDV strain (S272/16) has previously been identified as a member of the Europe-1 clade [37].

### 3.3. Organ and Cell Tropism of CaKV and CDV

**Figure 3:**
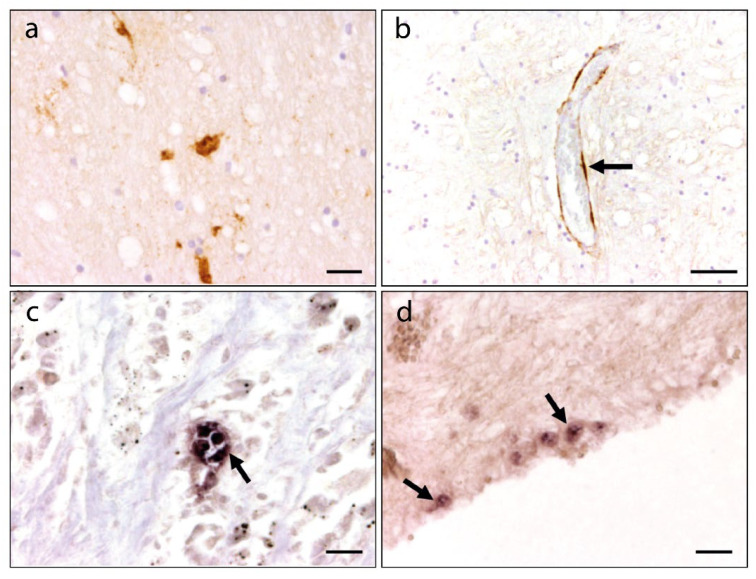
Immunohistochemical and in situ hybridization analysis of a canine-morbillivirus-infected fox co-infected with canine kobuvirus. (**a**) CDV antigen is detected by immunohistochemistry in the white matter of the cerebrum. (**b**) CDV antigen in endothelial cells of the cerebrum. (**c**) CaKV nucleic acids detected in epithelial cells of the stomach. (**d**) CaKV nucleic acids detected by in situ hybridization in ependymal cells of the cerebrum (arrows). Scale bar (**a**–**d**), 20 µm.

The organ and cell tropism of CDV and CaKV was investigated using a panel of formalin-fixed tissue samples and RNA extracted from different frozen tissue samples. Immunohistochemical staining showed the presence of CDV antigen in neurons, ependymal cells, and endothelial cells in the cerebrum (Figure 3a,b), with infected cells also observed in the cerebellum and brain stem (Table 1). Extensive CDV infection was also present in peripheral tissues including lung, kidney, urinary bladder, and spleen tissues (Table 1). Available organ samples were also analysed for the presence of CDV RNA using a previ-ously published RT-PCR assay. Multiple tissues including brain, kidney, lung, spleen, urinary bladder, trachea, blood, and pulmonary, inguinal, and intestinal lymph nodes were positive for CDV (Table 2). The potential organ tropism of CaKV in carnivore species was investigated using in situ hybridization to determine the distribution of CaKV RNA on a cellular level. CaKV-specific nucleic acids were found within epithelial cells of the stomach (Figure 3c), in ependymal cells of the cerebrum (Figure 3d), and in necrotizing foci of the lung (Table 1). A complementary RT-qPCR assay was used to show the presence of CaKV RNA in the urinary bladder, trachea, brain, and pulmonary inguinal and intestinal lymph nodes (Table 2).

## 4. Discussion

Canine kobuvirus has been described as an enteric pathogen which can be detected in both asymptomatic and diarrheic dogs. However, the real importance of CaKV as an etiological agent of gastrointestinal disease remains unclear. In this study, we have characterized the complete genome sequence and both the cellular and tissue tropism of a strain of CaKV which was detected in a CDV-infected fox with catarrhal enteritis. Systemic CDV and CaKV infections were detected in peripheral tissues and the central nervous system of the fox. This confirms the findings of a previous study, showing extraintestinal CaKV in a puppy which was co-infected with CDV, canine adenovirus type 1, canine herpesvirus type 1, and canine parvovirus type 2 [38]. The higher prevalence of CaKV infection in juvenile dogs in comparison to adult animals has been suggested to be due to a deficiency in their immune response [16]. Thus, a hematogenic distribution of CaKV from the gastrointestinal tract into lung, CNS, urinary bladder, tonsil, and multiple lymph nodes of the infected fox may have been facilitated by the lack of an appropriate immune response as a result of the CDV co-infection. Morbillivirus infections are known to induce severe generalized immunosuppression and modulation [39], which can contribute to secondary bacterial or viral infections [40,41]. This may occur as a result of direct infection of tissue-resident and circulating B-and T-cells [42]. The observed histologic lesions in the lung, urinary tract, and CNS represent common findings in CDV-infected animals [39]. Thus, it can be assumed that the observed systemic CaKV infection was enhanced by a CDV co-infection and associated immunosuppression. However, no correlation between the presence of CaKV RNA and histological lesions could be demonstrated. The detection of CaKV-specific nucleic acids in cerebral ependymal cells substantiates this potential underlying effect. With respect to the entero- and neuropathogenic potential of CaKV in foxes and other wild carnivore species, further investigations are required, but these findings substantiate previous observations that CaKV is circulating in fox populations in Europe [13,19].

Investigations into the pathogenesis and host cell tropism of CaKV are of special interest in the context of its close evolutionary relationship to human AiV. Epidemiological studies into AiV are limited, but it is known that this virus has a worldwide distribution, as evidenced by the detection of viral RNA in sewage water worldwide [43,44,45] and by high seroprevalence rates in older adults [46,47,48]. Although AiV infection has been predominately detected in patients with diarrheic symptoms, the potential role of co-infecting pathogens and/or immunosuppression in enhancing systemic virus spread remains to be determined. In addition, the findings of this study suggest that further research into the capacity of kobuviruses to spread to the CNS of animals and humans is warranted, particularly in cases complicated by co-infections or co-morbidities. In summary, the present study describes the first comprehensive assessment of the tissue and cellular tropism of CaKV and highlights the necessity for further research into the pathogenesis of kobuviruses.

## Figures and Tables

**Table 1:** Detection of CDV antigen and CaKV RNA in tissues from a co-infected fox.

Sample Tissue	CDV IHC	CaKV ISH
Cerebrum	+	+
Brain stem	+	−
Cerebellum	+	−
Trachea	−	−
Lung	+	+
Spleen	+	−
Tonsil	−	−
Stomach	−	+
Heart	−	−
Liver	−	−
Intestine	−	−
Lymph node	−	−
Kidney	+	−
Bladder	+	−
Testicle	−	−

IHC, Immunohistochemistry; ISH, in situ hybridization; −, negative; +, positive.

**Table 2:** Assessment of tissue distribution of CDV and CaKV RNA in a co-infected fox.

Sample Tissue	CDV RT-PCR	CaKV RT-qPCR
Brain	+	+ (28)
Trachea	+	+ (27)
Lung	+	−
Spleen	+	−
Lung LN	+	+ (30)
Intestinal LN	+	+ (35)
Inguinal LN	+	+ (30)
Kidney	+	−
Blood	+	− (42)
Bladder	+	+ (29)

Ct values are shown in brackets; −, negative; +, positive; * negative result, Ct > 35; positive result, Ct ≤ 35.

## Data Availability

The sequencing data presented in this study are available in GenBank, accession nos. MN337880 (Canine kobuvirus strain S272/16) and MN267061 (Canine distemper virus strain S272/16).
